# Heparin Differentially Regulates the Expression of Specific miRNAs in Mesenchymal Stromal Cells

**DOI:** 10.3390/ijms252312589

**Published:** 2024-11-23

**Authors:** Michaela Oeller, Tanja Schally, Georg Zimmermann, Wanda Lauth, Katharina Schallmoser, Eva Rohde, Sandra Laner-Plamberger

**Affiliations:** 1 Department for Transfusion Medicine, University Hospital of Salzburg (SALK), Paracelsus Medical University (PMU) Salzburg, Muellner Hauptstraße 48, 5020 Salzburg, Austria; m.oeller@salk.at (M.O.); k.schallmoser@salk.at (K.S.); e.rohde@salk.at (E.R.); 2 GMP Laboratory, PMU Salzburg, Strubergasse 21, 5020 Salzburg, Austria; tanja.schally@pmu.ac.at; 3 Team Biostatistics and Big Medical Data, IDA Lab Salzburg, PMU Salzburg, Strubergasse 16, 5020 Salzburg, Austria; georg.zimmermann@plus.ac.at (G.Z.); wanda.lauth@pmu.ac.at (W.L.); 4 Research Program Biomedical Data Science, PMU Salzburg, Strubergasse 16, 5020 Salzburg, Austria; 5 Department of Artificial Intelligence and Human Interfaces, Faculty of Digital and Analytical Sciences, Paris Lodron University Salzburg, Jakob Haringer Straße 2, 5020 Salzburg, Austria

**Keywords:** heparin, non-coding RNAs, miRNAs, expression, stromal cells, MSCs, human platelet lysate

## Abstract

In regenerative medicine, stromal cells are supposed to play an important role by modulating immune responses and differentiating into various tissue types. The aim of this study was to investigate the influence of heparin, frequently used as an anticoagulant in human platelet lysate (HPL)-supplemented cell cultures, on the expression of non-coding RNA species, particularly microRNAs (miRNA), which are pivotal regulators of gene expression. Through genomic analysis and quantitative RT-PCR, we assessed the differential impact of heparin on miRNA expression in various stromal cell types, derived from human bone marrow, umbilical cord and white adipose tissue. Our results demonstrate that heparin significantly alters miRNA expression, with distinct up- and downregulation patterns depending on the original tissue source of human stromal cells. Furthermore, our analyses indicate that these heparin-induced alterations in miRNA expression profiles influence critical cellular processes, including proliferation, apoptosis and differentiation. In conclusion, our study highlights that heparin not only fulfills its primary role as an efficient anticoagulant but can also modulate important regulatory pathways in stromal cells by influencing miRNA expression. This may alter cellular properties and thus influence stromal cell-based therapeutic applications in regenerative medicine.

## 1. Introduction

Stromal cells, frequently termed mesenchymal stromal/stem cells (MSCs), play an important role as cell-based therapeutics in regenerative medicine. Due to their ability to differentiate into various cell types and modulate immune responses, they offer potential treatment options for diverse disease conditions, such as degenerative bone diseases, cardiovascular disorders, wound healing and autoimmune diseases, and are of great value for the development of therapies for medical conditions that currently lack effective treatments [1,2,3]. Basic research and clinical trials utilizing stromal cells of different tissue sources such as bone marrow (BM), umbilical cord (UC) and white adipose tissue (WAT) have shown promising results for different diseases, highlighting the potential of stromal cells to efficiently promote tissue repair and regeneration [4,5].

Optimizing cell culture conditions to support the proliferation and differentiation of stromal cells is a key primary step for the efficient production of stromal cell-based therapeutics. Traditionally, animal-derived components like fetal bovine serum (FBS) are still used in cell culture media [6]. However, the quest for safer, more ethical and effective alternatives has led to the adoption of human platelet lysate (HPL) as a promising substitute [7,8]. HPL is rich in growth factors and cytokines and even promotes stromal cell proliferation better than animal-derived components independent of the preparation mode [7,9,10]. Since HPL-borne fibrinogen leads to the gelation of culture medium, the addition of an anticoagulant substance, usually the sulfated glycosaminoglycan heparin, is required [11]. Beyond its anticoagulant properties, different studies indicate that heparin may play a multifaceted role in cellular processes, including the modulation of protein-coding gene expression patterns [12,13,14,15,16]. This underscores heparin’s potential as a critical factor in stromal cell culture systems for therapeutic applications.

MicroRNAs (miRNAs) are small, single-stranded non-coding RNA molecules with an average size of 22 nucleotides that are abundant in different human and mammalian cell types [17,18,19]. They were shown to be crucial for the regulation of gene expression at the post-transcriptional level acting via mRNA degradation and/or translational repression in the cytoplasm, but also at the nuclear level by miRNA-guided control of transcription, mRNA export and mRNA splicing events [20,21,22,23]. The importance of miRNAs in gene regulation is emphasized by data indicating that up to 60% of the human protein-coding genome is regulated by miRNAs [24]. In addition to protein-coding genes, heparin may also influence the expression of certain miRNAs [25,26]. However, global expression data on this topic are still scarce, particularly regarding stromal cells. Even though miRNAs are suggested not to be master regulators of gene expression, but are rather fine tuners of transcriptional programs [27], their regulation by heparin may have an important influence on cell properties and thus affect also efficacy of stromal cell-based therapeutics.

This study aims to examine the multifaceted role of heparin in HPL-supplemented stromal cell cultures in more detail, focusing on its impact on miRNA expression. Through comprehensive genomic analyses, our data unveil the breadth of heparin’s influence on miRNA expression profiles across different stromal cell types. Our findings offer a new perspective on the use of heparin in cell culture, corroborating data indicating that its role extends beyond mere anticoagulation to encompass the modulation of key regulatory molecules such as miRNAs.

## 2. Results

### 2.1. The Expression of Different Non-Coding RNA Species Is Differentially Regulated in Stromal Cells by Heparin

**Figure 1:**
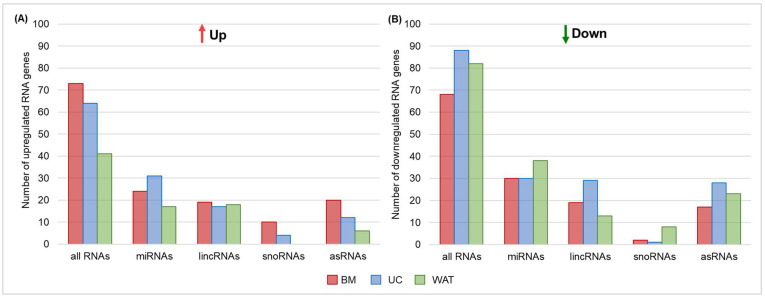
Heparin significantly regulates the expression of different RNA genes: miRNAs, lincRNAs, snoRNAs and asRNAs. Shown are numbers of significantly (*p* < 0.05) upregulated (≥1.2 fold) (**A**) or downregulated (≤−1.2 fold) (**B**) RNA genes in BM-, UC- or WAT-derived stromal cells (*n* = 3 for each tissue source) as indicated.

As shown previously, heparin significantly affects the expression of protein-coding genes in different stromal cell types [12,13]. Our whole genome expression analysis revealed, that not only protein-coding genes [13] but also the expression of non-coding RNA genes is significantly affected by heparin. In summary, the expression of 416 different RNA genes, including miRNAs, long intergenic non-coding RNAs (lincRNAs), small nucleolar RNAs (snoRNAs) and antisense-RNAs (asRNAs) were significantly regulated in BM-, UC- and WAT-derived stromal cells by the presence of heparin in culture medium. As shown in Figure 1A,B, 178 RNA genes were up- and 238 were downregulated (≥1.2 fold or ≤−1.2, *p* < 0.05), with miRNAs being the group of RNA genes that were significantly regulated most frequently by number.

### 2.2. Heparin Influences the Expression of miRNAs Differentially in a Cell Source Depending Manner

**Figure 2:**
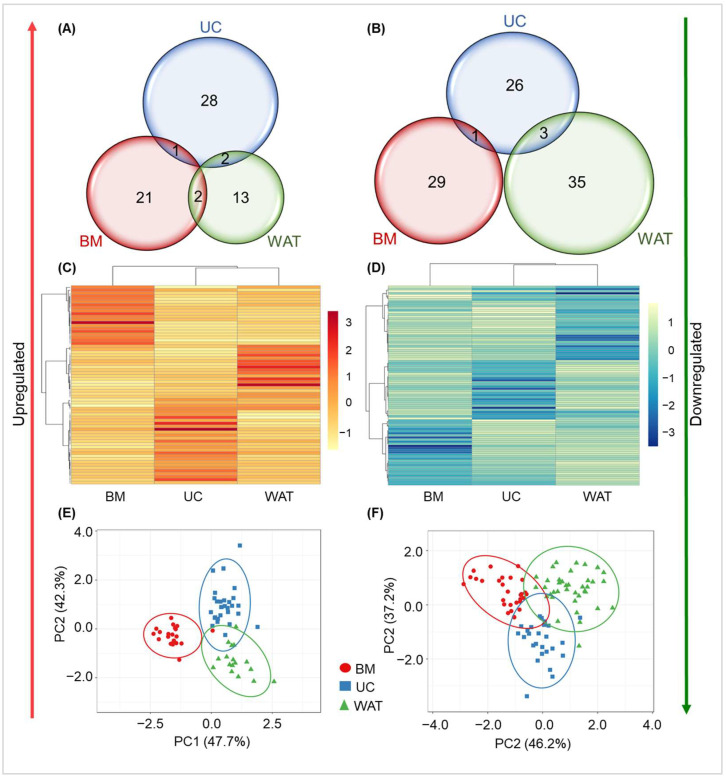
miRNA gene expression profiling in BM-, UC- or WAT-derived stromal cells cultured with or without heparin. Venn diagrams show the number of genes that were significantly up- (**A**) or downregulated (**B**) by heparin (fold change ≥ 1.2 or ≤−1.2 in three biological replicates for each tissue source, *p* < 0.05). Heat maps illustrating heparin-induced source-dependent upregulation (**C**) or downregulation (**D**) of miRNA gene expression. Principal component analysis (PCA) for upregulated (**E**) and downregulated (**F**) genes applying singular value decomposition with imputation. Prediction ellipses show that the miRNA gene expression profile of an additional stromal cell donor will fall into the corresponding ellipse with a probability of >95%.

Since miRNAs are known to be pivotal players in gene regulation [20,21,22], we focused on this type of RNA and analyzed the expression profiles of miRNAs in the different stromal cell types more closely. As already reported for protein-coding genes [13], we found that the expression of miRNA genes is affected differentially in a cell source-dependent manner. UC-derived stromal cells showed the highest number of miRNA genes upregulated by heparin with 31 genes compared to 24 genes in BM-, and 17 genes in WAT-derived stromal cells. Interestingly, none of the genes were commonly upregulated by heparin in all three cell types and only a few genes were significantly upregulated by heparin in two of the three tissue sources (BM and UC: MIR326; BM and WAT: MIR2909 and MIR4675; UC and WAT: MIR944 and MIR1912) (Figure 2A). We also found similar numbers of miRNA genes to be significantly downregulated by heparin in the three cell sources: 30 miRNAs for both UC- and BM-derived stromal cells and 38 miRNAs in WAT-derived stromal cells. As for upregulated miRNAs, no miRNA genes were significantly downregulated in all three cell sources and only a few miRNAs were significantly downregulated in two of the three sources (BM and UC: MIR488; UC and WAT: MIR376A1, MIRLET7F1 and MIR3619) (Figure 2B). A complete list of all significantly regulated miRNA target genes can be found in Supplementary Table S1. Our findings are also depicted in the heat maps in Figure 2C,D: miRNA genes significantly up- or downregulated by heparin in BM-derived stromal cells showed a significantly different expression pattern compared to WAT- or UC-derived stromal cells. The same was observed for the expression of miRNA genes significantly affected by heparin in UC- and WAT-derived stromal cells. The statistical significance of this finding was confirmed by a principal component analysis for upregulated (Figure 2E) and downregulated (Figure 2F) miRNA genes. The prediction ellipses indicate that the miRNA gene expression profile of heparin-exposed stromal cells of an additional donor would be inside the corresponding ellipse with a probability of >95%.

### 2.3. Quantitative RT-PCR Confirms Heparin-Induced Alteration in miRNA Gene Expression

**Figure 3:**
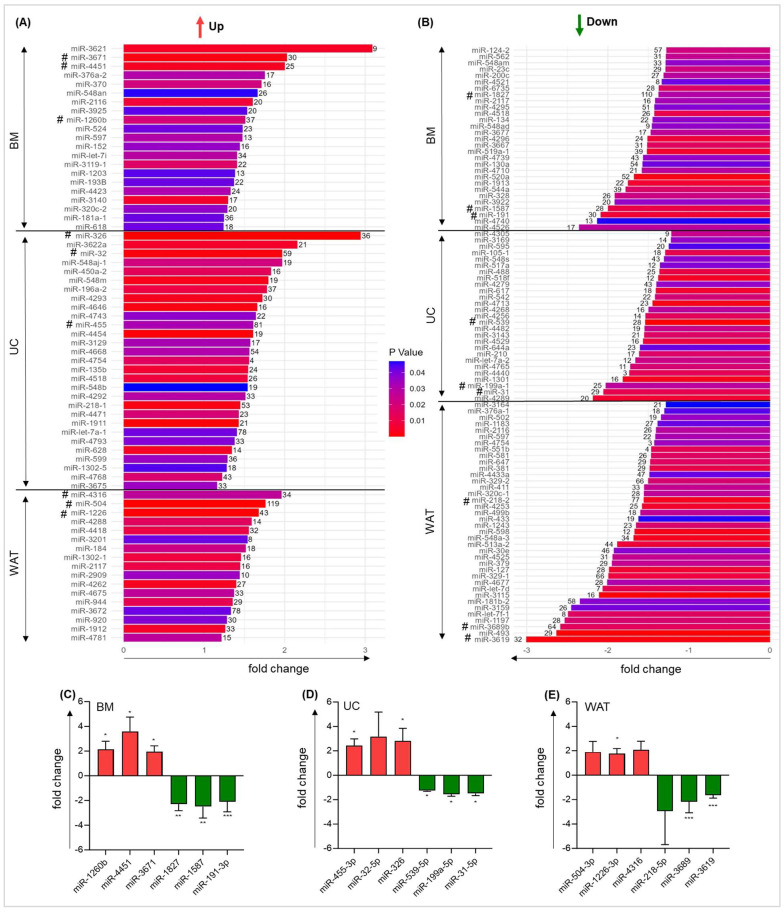
Heparin miRNA target gene expression. Top 15 significantly (*p* < 0.05) upregulated (≥1.2 fold change) (**A**) or downregulated (≤−1.2 fold change) (**B**) miRNA genes in response to heparin-culture according to fold change values obtained by microarray expression analysis (corresponding *p*-value depicted by color code as indicated) and to the number of known downstream target genes according to miRNet 2.0 tool (number shown next to the bar of each corresponding miRNA). Some miRNAs are among the top 15 in both rankings (according to fold change and according to the number of known downstream targets)—these miRNAs are depicted only once. miRNAs chosen for target verification are indicated by #. qRT-PCR corroborating heparin-induced gene expression changes of selected miRNA gene in (**C**) BM-, (**D**) UC- and (**E**) WAT-derived stromal cells. Data are mean fold change values of three biological triplicates for each tissue source measured in duplicates. Unpaired *t*-test was performed to identify statistically significant differences, * *p* < 0.05, ** *p* < 0.01, *** *p* < 0.001.

Next, we sorted significantly regulated miRNA genes according to their expression fold change values in response to heparin exposure and to the number of affected known downstream target genes according to miRNet 2.0 tool (https://www.mirnet.ca/miRNet/home.xhtml, accessed on 28 April 2024). The top 15 significantly up- and downregulated (*p* < 0.05) miRNAs for each tissue source according to fold change expression and the top 15 miRNAs according to the number of known downstream targets are shown in Figure 3A,B. For each stromal cell type, six miRNA target genes were chosen for target verification by qRT-PCR (Figure 3A,B). After total RNA isolation followed by polyadenylation, a specific Poly(T)-adapter primer, including a unique reverse primer sequence, was used for cDNA synthesis. Using a specific forward and a unique reverse primer, we could confirm the results of the global expression analysis for all miRNA targets chosen. We found significantly altered miRNA expression in response to heparin in BM-derived stromal cells (Figure 3C). We could also corroborate our findings for miRNAs in UC- and WAT-derived stromal cells, although not all findings were statistically significant (Figure 3D,E).

### 2.4. Heparin-Affected miRNA Target Gene Expression Influences Different Signaling Pathways and Cellular Processes

**Figure 4:**
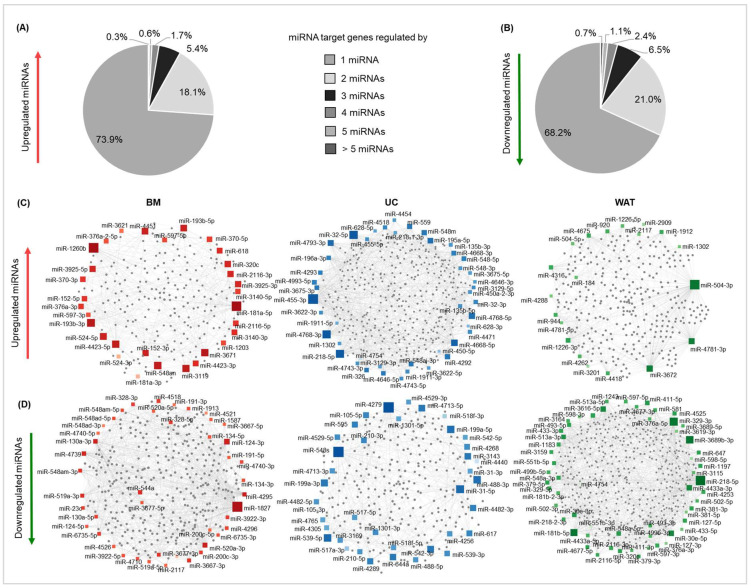
Heparin-regulated miRNAs affect multiple target genes. According to miRNet 2.0 (https://www.mirnet.ca/miRNet/home.xhtml, accessed on 28 April 2024), heparin-up- (**A**) and -downregulated (**B**) miRNAs target several hundred to more than a thousand downstream genes. The majority of genes are affected by only one miRNA (mean value for all stromal cell sources: upregulated: 73.9%, downregulated: 68.2%), while more than one-fourth of genes are targeted by multiple miRNAs (numbers of miRNAs as indicated). Force atlas of upregulated (**C**) and downregulated (**D**) miRNAs, depicting their downstream target gene network. Diagrams were created using miRNet 2.0 tool with the following filter settings: degree cutoff for all network nodes = 1; minimum network displayed. Each rectangle represents one miRNA, with the size and the color of the rectangle correlating to its number of downstream target genes (grey dots): larger and darker rectangles indicate a higher number of downstream targets. For clarity, only downstream targets that are regulated by at least two miRNAs are displayed.

According to miRNet 2.0 tool, significantly heparin-regulated miRNAs are known to be involved in the regulation of several hundred to more than a thousand downstream target genes, depending on the stromal cell source. The majority of downstream target genes are regulated by one of the heparin-regulated miRNAs (73.9% for upregulated miRNAs and 68.2% for downregulated miRNAs, average value for all tissue sources). However, more than one-fourth of target genes (26.1% targets of upregulated miRNAs, 31.7% targets of downregulated miRNAs, as average for all tissue sources) are regulated by two or more miRNAs (Figure 4A,B). The downstream target gene network of heparin-regulated miRNAs is shown as a force atlas for each tissue source in Figure 4C,D. Multiple network connections may indicate that these miRNAs could be critical key regulators influencing different regulatory networks (BM: miR-1260, miR-181a, miR-1827; UC: miR-3675, miR-4279 and miR-548; WAT: miR-504, miR-218, miR-3689 and miR-329).

**Figure 5:**
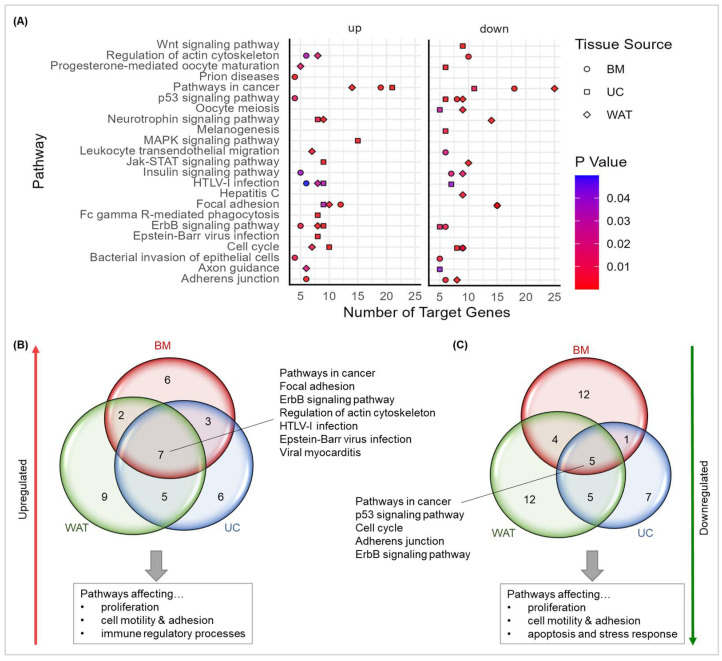
miRNet 2.0 functional enrichment analysis of pathways and cellular processes affected. (**A**) Pathways and cellular processes significantly influenced by heparin-induced regulation of miRNAs in three different stromal cell types. Data shown are numbers of target genes associated with pathways or cellular processes. Each stromal cell source is indicated as a symbol (BM: circle, UC: rectangle- and WAT-derived stromal cells: diamond). *p*-values for each source and pathway/cellular process are shown by color code as indicated. Common pathways and cellular processes influenced by heparin-upregulated miRNAs (**B**) and by heparin-downregulated miRNAs (**C**). Pathways commonly regulated by heparin-affected miRNAs in all stromal cell types are listed next to the Venn diagrams, the affected cellular processes are listed below.

Next, we performed a functional enrichment analysis using all significantly heparin-regulated miRNAs applying the miRNet 2.0 tool to reveal the pathways and cellular processes affected by heparin-induced alterations of miRNA gene expression. We found distinct, but also overlapping pathways and cellular processes for the three stromal cell sources. Genes of pathways and cellular processes being regulated by these miRNAs are depicted in Figure 5A. These findings are summarized in Figure 5B,C, emphasizing specificities and commonalities of the three different stromal cell types. The regulatory networks that were commonly upregulated are pathways and processes associated with proliferation (pathways in cancer, ErbB signaling pathway), cell motility and adhesion (focal adhesion, regulation of the actin cytoskeleton) and immune regulatory processes (pathways being associated with viral infections). Pathways generally affected by heparin-induced downregulation of miRNA target gene expression regarded proliferation (pathways in cancer, cell cycle), cell motility and adhesion (adherens junction) and the regulation of apoptosis and stress responses (p53 signaling pathway). We also found source-dependent effects regarding pathways: while the insulin signaling pathway was affected by up-and downregulated miRNAs in BM-derived stromal cells, it was downregulated in WAT-derived stromal cells and not among the significantly regulated pathways in stromal cells from UC. Jak-STAT signaling was upregulated in UC, but it was downregulated in WAT-derived stromal cells. MAPK signaling pathway was found to be significantly regulated in UC-derived stromal cells only. In summary, our findings point towards a well-orchestrated heparin-induced regulation of miRNA gene expression in a cell source-dependent manner that mainly affects cell proliferation, cell motility and adhesion, but also immune regulatory processes, apoptosis and stress responses.

## 3. Discussion

Ex vivo cultivation and proliferation of human stromal cells is an important prerequisite for stromal cell-based therapeutics, whereby great efforts are made to optimize safe, ethical and effective cell culture conditions. HPL-supplemented media enable humanized culture conditions, whereas the addition of heparin is required to avoid fibrin network formation and gelation of HPL-based culture media. For more than a century heparin has been known for its potent anticoagulant properties and has been used in daily clinical routine since 1935 [28]. Furthermore, it has been in use for HPL-based stromal cell culture systems for more than two decades. In 2013, it was reported that heparin significantly affected stromal cell proliferation and colony-forming capacity in a concentration-dependent manner [11]. The mechanical depletion of fibrinogen allowed us to study the effect of heparin on stromal cell proliferation in HPL-supplemented cell culture media in previous studies, indicating tissue source-dependent effects on proliferation and colony forming capacity, while the immunophenotype of stromal cells was not affected [13,29]. Furthermore, we and others showed that heparin significantly affects protein-coding gene expression patterns in stromal cells [12,13]. Regarding stromal cell differentiation, data are more divergent: some studies indicate a significant influence of heparin [15,30], but others did not observe such an effect [13,31].

In the present study, we explored the differential regulatory effects of low-dose heparin (2 IU/mL) on non-coding RNA species in HPL-based stromal cell culture, with a focus on miRNA expression. This concentration of heparin is required to prevent efficiently fibrinogen-induced gel formation of HPL and does not negatively affect proliferative capacities of the stromal cells used. Our data revealed a significant impact of heparin on miRNA expression, showing both upregulation and downregulation of specific miRNAs in different human stromal cell sources—bone marrow, umbilical cord and white adipose tissue. Notably, we focused on the global effects of heparin rather than individual changes. Thus, our study includes only miRNA expression changes in response to heparin that occurred in all three donors of each tissue source, excluding donor variability as far as possible. The differences in miRNA profiles between various stromal cell types suggest that heparin’s influence is not uniform but depends significantly on the cell source. This finding is well-aligned with our data regarding protein-coding genes [13] and other studies, which revealed divergent differentiation potential for stromal cells from different tissue sources, indicating that epigenetics is an important factor for stromal cell properties [32].

Applying the miRNet 2.0 tool, we examined which miRNA downstream target genes and thus which cellular processes and pathways are affected by heparin-induced alterations of miRNA expression. We identified high numbers of downstream target genes for each stromal cell type, which aligns with data indicating that a single miRNA may affect the expression patterns of several hundred target genes [33,34]. Our data further reveal that the majority of heparin-affected miRNA target genes are regulated by one miRNA, while more than one-fourth of downstream target genes are affected by multiple miRNAs. Again, each tissue source revealed distinct specific miRNAs with multiple downstream targets, which indicates that those heparin-regulated miRNAs may be critical key regulators of several factors affecting different regulatory networks [34]. Using a functional enrichment analysis conducted with miRNet 2.0, we found distinct, but also overlapping pathways and cellular processes being influenced by heparin-regulated miRNAs. Pathways associated with proliferation, cell motility and adhesion were significantly affected by both up- and downregulated miRNAs, pointing towards a well-orchestrated and balanced regulation of these cellular processes. Heparin-upregulated miRNAs were found to rather affect immune regulatory processes, while downregulated miRNAs influence apoptosis and stress responses. This is consistent with data from previous studies conducted in FBS- and HPL-supplemented culture conditions with and without heparin, where protein-coding gene profiling revealed the heparin-induced regulation of distinct signaling cascades involved in proliferation, cell adhesion, apoptosis and inflammation [12,13].

Our current data do not provide an explanation for how heparin induces the changes in miRNA expression. However, one possible explanation could be that heparin enhances the signaling of HPL-borne growth factors and cytokines by mimicking the role of glycosaminoglycans on cell surfaces, thus impacting cellular signaling as shown for receptors such as FGFR [35,36]. As demonstrated in our previous study [13], heparin is internalized by stromal cells. Once incorporated it may directly interfere with transcription factors [37], thus affecting downstream target gene expression.

Originally, miRNAs were supposed to elicit their effect by silencing the expression of target genes. However, there is increasing evidence indicating that miRNAs may also positively regulate gene expression, pointing to an even more complex nature of miRNAs in gene regulation [38,39]. Therefore, it is difficult to predict exactly the effect of heparin-induced miRNA expression alterations on cellular behavior. An important factor is the heparin concentration applied. As demonstrated by several studies, diverse concentrations of heparin may differentially affect gene expression and cellular properties: While low dose heparin affected stromal cell proliferation and differentiation [12,13], high concentrations inhibited stromal cell growth and increased senescence and cell size [11,12]. Therefore, it is important to note that our results apply to low doses of heparin (2 IU/mL) and that higher doses may change miRNA expression profiles to another extent and direction.

The present study has several strengths, but also limitations: Genomic analyses and quantitative RT-PCR represent a solid methodological framework, allowing thorough examination of heparin-induced alterations of miRNA expression in different stromal cells of several donors, thus ensuring reliability and reproducibility of the findings. Our data highlight heparin’s function beyond anticoagulation, providing evidence for its role as a modulator of miRNA expression. This function is crucial, as miRNAs play significant roles in regulating gene expression, influencing cell differentiation, proliferation and apoptosis, all key processes in regenerative medicine. By analyzing the effects of heparin on stromal cells derived from various tissues, our data corroborate our previous findings of tissue-specific responses to heparin [13]. These source-specific effects might be important for cell therapeutic approaches and their outcomes. However, the present study does not include a phenotype analysis of MSCs to support the significance of miRNA function. To investigate the effect of heparin on miRNAs in more detail, additional studies are required to compare the cellular phenotypes with or without heparin. In addition, while the present study outlines the in vitro effects of heparin on miRNA profiles, it does not provide in vivo data or clinical correlation. Further studies are clearly needed to verify how changes may affect disease models or even patient outcomes. Furthermore, our study focuses exclusively on heparin’s impact, potentially overlooking the synergistic or antagonistic effects that other components of HPL-based medium might exhibit. Future studies should consider the interaction between heparin and other factors to understand the milieu of HPL-based stromal cell culture. Taking into account the diverse nature of miRNA functions, the study’s focus on specific miRNAs that were most affected by heparin might neglect other miRNAs that could equally play significant roles in cellular function and thus affect the efficacy of cell-based therapeutic approaches.

In conclusion, our findings indicate that heparin plays a crucial role in modulating the expression of miRNAs, which are key modulators of gene expression. Our data provide further insight into the mechanisms by which heparin modifies stromal cell behavior, potentially guiding optimization of cell culture conditions and thus enhancing the therapeutic potential of stromal cell-based therapeutic approaches.

## 4. Materials and Methods

### 4.1. Ethical Statement

The present study was conducted in accordance with the Declaration of Helsinki and its later amendments. The use of human white adipose tissue and human umbilical cord tissue was approved by the ethical committee of the Federal State of Salzburg (vote numbers 415-E/1904/6-2015 and 415-E/1547/2-2012). Bone marrow samples were obtained from AllCells, (Alameda, CA, USA, https://allcells.com/research-grade-tissue-products/bone-marrow/, accessed on 22 March 2024). All donors of BM, WAT and UC tissues signed an informed consent concerning the use of the donated material for research purposes. Samples were processed anonymously to protect donor privacy.

### 4.2. Cell Isolation and Cell Culture Conditions

Three biological replicates were used for each stromal cell type and isolation was performed as described previously [13]. For isolation and propagation, alpha-modified Minimum Essentials Eagle’s Medium (α-MEM, Sigma Aldrich, St. Louis, MO, USA) supplemented (*v*/*v*) with 10% human platelet lysate (HPL, produced as described in [40,41]) and 5.5 mM (N2)-L-Alanyl-L-Glutamin (Dipeptiven, Fresenius Kabi, Graz, Austria) was used. HPL-supplemented α-MEM was mechanically fibrinogen-depleted as described previously [29] to avoid calcium-induced fibrinogen clot formation. For all isolation and cultivation steps, two different medium types were used: HPL-supplemented mechanically fibrinogen-depleted α-MEM (1) with heparin (2 IU/mL; Biochrom, Berlin, Germany) or (2) without heparin. The concentration of 2 IU/mL heparin was chosen for our experimental settings, as this amount is recommended [42] and thus frequently applied to efficiently avoid fibrinogen clot formation in HPL-supplemented media that are not mechanically fibrinogen depleted [9,11,29]. Higher heparin concentrations hamper cell proliferation (Supplementary Figure S1), which is also in line with previous studies [11]. Media were sterile filtrated before usage. Cells were cultured in Falcon T225 cell culture flasks (Corning Life Sciences, Tewksbury, MA, USA) at 37 °C and ambient air conditions. It is important to note that heparin-containing and heparin-free isolation and cultivation were performed completely separately from each other for each step, using the same source of biological material. For determining the proliferative behavior in response to different heparin concentrations, stromal cells (*n* = 3 for each tissue source) were cultured in mechanically fibrinogen-depleted HPL-supplemented medium (seeding density 100 cells/cm^2^ in 225 cm^2^ flasks in duplicates). Total cell counts were determined as described previously [10].

### 4.3. RNA Isolation, Determination of RNA Quality and Quantity and Whole Genome Expression Analysis

Total RNA isolation was performed using the High Pure RNA isolation kit (Roche Diagnostics, Rotkreuz, Switzerland) according to the manufacturer’s instructions. Quality and quantity of total RNA and miRNA were analyzed by applying the Agilent RNA 6000 Nano Kit and the Agilent Small RNA kit (both Agilent Technologies, Santa Clara, CA, USA) on an Agilent 2100 Bioanalyzer (Agilent Technologies) according to manufacturer’s protocol. RNA samples with an integrity number > 9 were used for gene expression analysis. Whole genome expression analysis was performed using the Affymetrix Human Gene 2.1 ST array (ThermoScientific—Affymetrix, Waltham, MA, USA) and three biological replicates for each BM-, UC- and WAT-derived stromal cell. Labeling of RNA samples, hybridizations and array chip scans were carried out at the Core Facility for Fluorescent Bioanalytics of the University of Regensburg, Germany.

### 4.4. Poly(A)-Tailing and cDNA Synthesis

For polyadenylation of total RNA the Poly(A) Tailing Kit (Applied Biosystems, Waltham, MA, USA) was used according to manufacturer’s instructions. Subsequently, free nucleotides were removed by completing a filtration step by applying the High Pure RNA isolation kit (Roche Diagnostics). cDNA was synthesized from 2 μg purified total RNA with Superscript II (Invitrogen) according to the manufacturer’s protocol using a Poly(T)-adapter primer (for primer sequence see Supplementary Table S2). In addition to the Poly(T) sequence, the Poly(T)-adapter primer contains a unique linker sequence, which was used as a unique reverse primer sequence for linear poly(A)-tailed quantitative real-time PCR (qRT-PCR).

### 4.5. miRNA Primer Design and qRT-PCR of Mature miRNAs

For miRNA primer design, the sRNAPrimerDB primer design service tool for small non-codingRNAs (http://www.srnaprimerdb.com, last accessed on 22 March 2024), was used [43]. qRT-PCR analysis of mature miRNAs is based on polyadenylation and the use of a unique reverse primer in combination with specific forward primer sequences [44,45,46]. The analysis was conducted on a LightCycler 480 II using the LightCycler 480 SYBR Green I Master reagent (both Roche Diagnostics) according to manufacturer’s instructions (thermocycler program: 95 °C for 1 min, 50 cycles (95 °C for 1 min, 60 °C for 30 s, 72 °C for 1 min), final extension at 72 °C for 10 min). Putative miRNA target genes to be confirmed by qRT-PCR were selected according to their fold expression change in response to heparin treatment, the corresponding *p*-value and their ranking according to the number of genes they target. For normalization of sample material, human SNORD44 and SNORD48 RNA genes were used, as suggested previously [47,48]. Both SNORD44 and SNORD48 showed stable expression profiles on all our microarrays conducted, without expression changes in response to heparin-containing culture conditions. Data analysis was conducted as described previously [49]. For qRT-PCR primer sequences see Supplementary Table S2.

### 4.6. Data Analysis and Statistics

Data analysis of Affymetrix Human Gene 2.1 ST array was conducted using R [50] with Bioconductor and ClusterProfiler add-on packages as described previously [13]. Visualizations regarding *p*-values, number of targets and fold changes were performed using the R-package ggplot2 within the statistical software R (Version 4.3.2) [51]. miRNA genes with an adjusted *p*-value of ≤0.05 and an absolute fold change of ≥1.2 or ≤−1.2 were considered as significantly differentially expressed. ClustVis tool was applied to create heatmaps and do the principal component analysis [52]. miRNet 2.0 was employed for the identification of putative downstream target genes of all heparin-regulated miRNAs (listed in Supplementary Table S1) including the functional enrichment analysis. For statistical analysis other than gene expression profiling GraphPad Prism 10 (GraphPad Software, Boston, MA, USA) was used. D’Agostino and Pearson omnibus normality test were performed to test for Gaussian distribution. For data analysis one-way ANOVA or unpaired *t*-tests were conducted, with *p* < 0.05 being considered as significant.

## Data Availability

The datasets generated during the present study are available from the corresponding author upon reasonable request.

## Supplementary Materials

The following supporting information can be downloaded at https://www.mdpi.com/article/10.3390/ijms252312589/s1.
