# Supplementary material for: Heparin Differentially Regulates the Expression of Specific miRNAs in Mesenchymal Stromal Cells
Source: Int J Mol Sci. 2024 Nov 23;25(23):12589. doi: 10.3390/ijms252312589 (PMC11641817; doi:10.3390/ijms252312589)
Supplement: Supplementary file 1 [file ijms-25-12589-s001.zip › Supplementary_FigureS1.pdf]

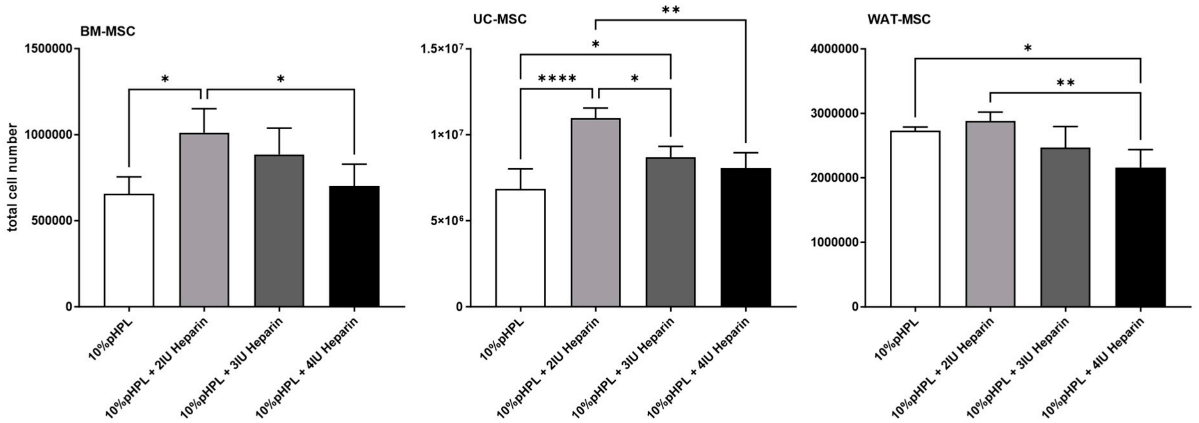

Supplementary Figure S1: Stromal cell proliferation in mechanically fibrinogen depleted HPL-medium in response to different heparin concentrations (2, 3 and 4 IU/mL). For each source three biological replicates were measured in duplicates, * $p < 0.05$, ** $p < 0.01$, **** $p < 0.0001$.
