# Supplementary material for: Heparin Differentially Regulates the Expression of Specific miRNAs in Mesenchymal Stromal Cells
Source: Int J Mol Sci. 2024 Nov 23;25(23):12589. doi: 10.3390/ijms252312589 (PMC11641817; doi:10.3390/ijms252312589)
Supplement: Supplementary file 1 [file ijms-25-12589-s001.zip › Supplementary_TableS1.pdf]

	MSC source	miRNA symbol	miRNA name	Link to mirbase (https://mirbase.org/)	Fold change (mean of n = 3)	p-value
Up	WAT	MIR4316	hsa-miR-4316	https://mirbase.org/hairpin/MI0015845	1,96	0,028
	WAT	MIR504	hsa-miR-504	https://mirbase.org/hairpin/MI0003189	1,76	0,000
	WAT	MIR1226	hsa-miR-1226	https://mirbase.org/hairpin/MI0006313	1,68	0,040
	WAT	MIR4288	hsa-miR-4288	https://mirbase.org/hairpin/MI0015896	1,59	0,015
	WAT	MIR4418	hsa-miR-4418	https://mirbase.org/hairpin/MI0016754	1,56	0,010
	WAT	MIR3201	hsa-miR-3201	https://mirbase.org/hairpin/MI0014250	1,54	0,043
	WAT	MIR184	hsa-miR-184	https://mirbase.org/hairpin/MI0000481	1,52	0,024
	WAT	MIR1302-1	hsa-miR-1302-1	https://mirbase.org/hairpin/MI0006362	1,46	0,009
	WAT	MIR2117	hsa-miR-2117	https://mirbase.org/hairpin/MI0010636	1,45	0,010
	WAT	MIR2909	hsa-miR-2909	https://mirbase.org/hairpin/MI0013083	1,44	0,036
	WAT	MIR4262	hsa-miR-4262	https://mirbase.org/hairpin/MI0015872	1,40	0,004
	WAT	MIR4675	hsa-miR-4675	https://mirbase.org/hairpin/MI0017306	1,37	0,028
	WAT	MIR944	hsa-miR-944	https://mirbase.org/hairpin/MI0005769	1,35	0,013
	WAT	MIR3672	hsa-miR-3672	https://mirbase.org/hairpin/MI0016073	1,33	0,044
	WAT	MIR920	hsa-miR-920	https://mirbase.org/hairpin/MI0005712	1,28	0,041
	WAT	MIR1912	hsa-miR-1912	https://mirbase.org/hairpin/MI0008333	1,26	0,005
	WAT	MIR4781	hsa-miR-4781	https://mirbase.org/hairpin/MI0017426	1,22	0,028
Down	WAT	MIR3164	hsa-miR-3164	https://mirbase.org/hairpin/MI0014194	-1,29	0,047
	WAT	MIR376A1	hsa-miR-376a-1	https://mirbase.org/hairpin/MI0000784	-1,30	0,044
	WAT	MIR502	hsa-miR-502	https://mirbase.org/hairpin/MI0003186	-1,35	0,036
	WAT	MIR1183	hsa-miR-1183	https://mirbase.org/hairpin/MI0006276	-1,39	0,042
	WAT	MIR2116	hsa-miR-2116	https://mirbase.org/hairpin/MI0010635	-1,41	0,028
	WAT	MIR597	hsa-miR-597	https://mirbase.org/hairpin/MI0003609	-1,42	0,032
	WAT	MIR4754	hsa-miR-4754	https://mirbase.org/hairpin/MI0017394	-1,43	0,035
	WAT	MIR551B	hsa-miR-551b	https://mirbase.org/hairpin/MI0003575	-1,47	0,013
	WAT	MIR581	hsa-miR-581	https://mirbase.org/hairpin/MI0003588	-1,47	0,015
	WAT	MIR647	hsa-miR-647	https://mirbase.org/hairpin/MI0003662	-1,48	0,015
	WAT	MIR381	hsa-miR-381	https://mirbase.org/hairpin/MI0000789	-1,49	0,010

	WAT	MIR4433A	hsa-miR-4433a	https://mirbase.org/hairpin/MI0016773	-1,49	0,042
	WAT	MIR329-2	hsa-miR-329-2	https://mirbase.org/hairpin/MI0001726	-1,50	0,019
	WAT	MIR411	hsa-miR-411	https://mirbase.org/hairpin/MI0003675	-1,55	0,025
	WAT	MIR320C1	hsa-miR-320c-1	https://mirbase.org/hairpin/MI0003778	-1,56	0,027
	WAT	MIR218-2	hsa-miR-218-2	https://mirbase.org/hairpin/MI0000295	-1,56	0,034
	WAT	MIR4253	hsa-miR-4253	https://mirbase.org/hairpin/MI0015860	-1,58	0,005
	WAT	MIR499B	hsa-miR-499b	https://mirbase.org/hairpin/MI0017396	-1,60	0,026
	WAT	MIR433	hsa-miR-433	https://mirbase.org/hairpin/MI0001723	-1,62	0,046
	WAT	MIR1243	hsa-miR-1243	https://mirbase.org/hairpin/MI0006373	-1,65	0,018
	WAT	MIR598	hsa-miR-598	https://mirbase.org/hairpin/MI0003610	-1,67	0,003
	WAT	MIR548A3	hsa-miR-548a-3	https://mirbase.org/hairpin/MI0003612	-1,68	0,003
	WAT	MIR513A2	hsa-miR-513a-2	https://mirbase.org/hairpin/MI0003192	-1,89	0,008
	WAT	MIR30E	hsa-miR-30e	https://mirbase.org/hairpin/MI0000749	-1,93	0,038
	WAT	MIR4525	hsa-miR-4525	https://mirbase.org/hairpin/MI0016892	-1,94	0,025
	WAT	MIR379	hsa-miR-379	https://mirbase.org/hairpin/MI0000787	-1,95	0,025
	WAT	MIR127	hsa-miR-127	https://mirbase.org/hairpin/MI0000472	-1,99	0,006
	WAT	MIR329-1	hsa-miR-329-1	https://mirbase.org/hairpin/MI0001725	-1,99	0,009
	WAT	MIR4677	hsa-miR-4677	https://mirbase.org/hairpin/MI0017308	-2,01	0,031
	WAT	MIRLET7D	hsa-miR-let-7d	https://mirbase.org/hairpin/MI0000065	-2,07	0,012
	WAT	MIR3115	hsa-miR-3115	https://mirbase.org/hairpin/MI0014127	-2,11	0,001
	WAT	MIR181B2	hsa-miR-181b-2	https://mirbase.org/hairpin/MI0000683	-2,35	0,038
	WAT	MIR3159	hsa-miR-3159	https://mirbase.org/hairpin/MI0014188	-2,45	0,041
	WAT	MIRLET7F1	hsa-miR-let-7f-1	https://mirbase.org/hairpin/MI0000067	-2,49	0,015
	WAT	MIR1197	hsa-miR-1197	https://mirbase.org/hairpin/MI0006656	-2,53	0,018
	WAT	MIR3689B	hsa-miR-3689b	https://mirbase.org/hairpin/MI0016411	-2,59	0,014
	WAT	MIR493	hsa-miR-493	https://mirbase.org/hairpin/MI0003132	-2,64	0,000
	WAT	MIR3619	hsa-miR-3619	https://mirbase.org/hairpin/MI0016009	-3,01	0,001
Up	UC	MIR326	hsa-miR-326	https://mirbase.org/hairpin/MI0000808	2,94	0,000
	UC	MIR3622A	hsa-miR-3622a	https://mirbase.org/hairpin/MI0016013	2,16	0,005

	UC	MIR32	hsa-miR-32	https://mirbase.org/hairpin/MI0000090	1,97	0,001
	UC	MIR548AJ1	hsa-miR-548aj-1	https://mirbase.org/hairpin/MI0016814	1,97	0,022
	UC	MIR450A2	hsa-miR-450a-2	https://mirbase.org/hairpin/MI0003187	1,83	0,014
	UC	MIR548M	hsa-miR-548m	https://mirbase.org/hairpin/MI0006400	1,80	0,003
	UC	MIR196A2	hsa-miR-196a-2	https://mirbase.org/hairpin/MI0000279	1,78	0,009
	UC	MIR4293	hsa-miR-4293	https://mirbase.org/hairpin/MI0015826	1,72	0,003
	UC	MIR4646	hsa-miR-4646	https://mirbase.org/hairpin/MI0017273	1,66	0,002
	UC	MIR4743	hsa-miR-4743	https://mirbase.org/hairpin/MI0017381	1,64	0,039
	UC	MIR455	hsa-miR-455	https://mirbase.org/hairpin/MI0003513	1,61	0,033
	UC	MIR4454	hsa-miR-4454	https://mirbase.org/hairpin/MI0016800	1,61	0,001
	UC	MIR3129	hsa-miR-3129	https://mirbase.org/hairpin/MI0014146	1,57	0,034
	UC	MIR4668	hsa-miR-4668	https://mirbase.org/hairpin/MI0017298	1,56	0,032
	UC	MIR4754	hsa-miR-4754	https://mirbase.org/hairpin/MI0017394	1,56	0,015
	UC	MIR135B	hsa-miR-135b	https://mirbase.org/hairpin/MI0000810	1,55	0,006
	UC	MIR4518	hsa-miR-4518	https://mirbase.org/hairpin/MI0016884	1,54	0,008
	UC	MIR548B	hsa-miR-548b	https://mirbase.org/hairpin/MI0003596	1,54	0,049
	UC	MIR4292	hsa-miR-4292	https://mirbase.org/hairpin/MI0015897	1,51	0,030
	UC	MIR218-1	hsa-miR-218-1	https://mirbase.org/hairpin/MI0000294	1,45	0,002
	UC	MIR4471	hsa-miR-4471	https://mirbase.org/hairpin/MI0016822	1,43	0,020
	UC	MIR1911	hsa-miR-1911	https://mirbase.org/hairpin/MI0008332	1,43	0,001
	UC	MIRLET7A1	hsa-miR-let-7a-1	https://mirbase.org/hairpin/MI0000060	1,41	0,043
	UC	MIR4793	hsa-miR-4793	https://mirbase.org/hairpin/MI0017440	1,38	0,041
	UC	MIR628	hsa-miR-628	https://mirbase.org/hairpin/MI0003642	1,34	0,004
	UC	MIR599	hsa-miR-599	https://mirbase.org/hairpin/MI0003611	1,29	0,037
	UC	MIR1302-5	hsa-miR-1302-5	https://mirbase.org/hairpin/MI0006366	1,28	0,046
	UC	MIR4768	hsa-miR-4768	https://mirbase.org/hairpin/MI0017409	1,22	0,019
	UC	MIR3675	hsa-miR-3675	https://mirbase.org/hairpin/MI0016076	1,16	0,036
Down	UC	MIR4305	hsa-miR-4305	https://mirbase.org/hairpin/MI0015835	-1,22	0,024
	UC	MIR3169	hsa-miR-3169	https://mirbase.org/hairpin/MI0014200	-1,22	0,036
	UC	MIR595	hsa-miR-595	https://mirbase.org/hairpin/MI0003607	-1,25	0,044

	UC	MIR105-1	hsa-miR-105-1	https://mirbase.org/hairpin/MI0000111	-1,29	0,012
	UC	MIR548S	hsa-miR-548s	https://mirbase.org/hairpin/MI0014141	-1,31	0,037
	UC	MIR517A	hsa-miR-517a	https://mirbase.org/hairpin/MI0003161	-1,36	0,039
	UC	MIR488	hsa-miR-488	https://mirbase.org/hairpin/MI0003123	-1,36	0,010
	UC	MIR518F	hsa-miR-518f	https://mirbase.org/hairpin/MI0003154	-1,38	0,004
	UC	MIR4279	hsa-miR-4279	https://mirbase.org/hairpin/MI0015887	-1,40	0,036
	UC	MIR617	hsa-miR-617	https://mirbase.org/hairpin/MI0003631	-1,41	0,007
	UC	MIR542	hsa-miR-542	https://mirbase.org/hairpin/MI0003686	-1,42	0,020
	UC	MIR4713	hsa-miR-4713	https://mirbase.org/hairpin/MI0017347	-1,45	0,002
	UC	MIR4268	hsa-miR-4268	https://mirbase.org/hairpin/MI0015874	-1,50	0,028
	UC	MIR4256	hsa-miR-4256	https://mirbase.org/hairpin/MI0015855	-1,54	0,014
	UC	MIR539	hsa-miR-539	https://mirbase.org/hairpin/MI0003514	-1,54	0,020
	UC	MIR4482	hsa-miR-4482	https://mirbase.org/hairpin/MI0016843	-1,55	0,024
	UC	MIR3143	hsa-miR-3143	https://mirbase.org/hairpin/MI0014167	-1,55	0,016
	UC	MIR4529	hsa-miR-4529	https://mirbase.org/hairpin/MI0016896	-1,57	0,009
	UC	MIR644A	hsa-miR-644a	https://mirbase.org/hairpin/MI0003659	-1,61	0,042
	UC	MIR210	hsa-miR-210	https://mirbase.org/hairpin/MI0000286	-1,61	0,017
	UC	MIRLET7A2	hsa-miR-let-7a-2	https://mirbase.org/hairpin/MI0000061	-1,66	0,023
	UC	MIR4765	hsa-miR-4765	https://mirbase.org/hairpin/MI0017406	-1,72	0,019
	UC	MIR4440	hsa-miR-4440	https://mirbase.org/hairpin/MI0016783	-1,74	0,013
	UC	MIR1301	hsa-miR-1301	https://mirbase.org/hairpin/MI0003815	-1,82	0,003
	UC	MIR199A1	hsa-miR-199a-1	https://mirbase.org/hairpin/MI0000242	-2,03	0,028
	UC	MIR31	hsa-miR-31	https://mirbase.org/hairpin/MI0000089	-2,06	0,012
	UC	MIR4289	hsa-miR-4289	https://mirbase.org/hairpin/MI0015898	-2,19	0,006
Up	BM	MIR3621	hsa-miR-3621	https://mirbase.org/hairpin/MI0016012	3,09	0,004
	BM	MIR3671	hsa-miR-3671	https://mirbase.org/hairpin/MI0016072	2,03	0,002
	BM	MIR4451	hsa-miR-4451	https://mirbase.org/hairpin/MI0016797	2,00	0,004
	BM	MIR376A2	hsa-miR-376a-2	https://mirbase.org/hairpin/MI0003529	1,75	0,031
	BM	MIR370	hsa-miR-370	https://mirbase.org/hairpin/MI0000778	1,71	0,022

	BM	MIR548AN	hsa-miR-548an	https://mirbase.org/hairpin/MI0016907	1,66	0,048
	BM	MIR2116	hsa-miR-2116	https://mirbase.org/hairpin/MI0010635	1,60	0,008
	BM	MIR3925	hsa-miR-3925	https://mirbase.org/hairpin/MI0016433	1,53	0,045
	BM	MIR1260B	hsa-miR-1260b	https://mirbase.org/hairpin/MI0014197	1,51	0,021
	BM	MIR524	hsa-miR-524	https://mirbase.org/hairpin/MI0003160	1,48	0,043
	BM	MIR597	hsa-miR-597	https://mirbase.org/hairpin/MI0003609	1,48	0,031
	BM	MIR152	hsa-miR-152	https://mirbase.org/hairpin/MI0000462	1,44	0,037
	BM	MIRLET7I	hsa-miR-let-7i	https://mirbase.org/hairpin/MI0000434	1,41	0,030
	BM	MIR3119-1	hsa-miR-3119-1	https://mirbase.org/hairpin/MI0014134	1,41	0,017
	BM	MIR1203	hsa-miR-1203	https://mirbase.org/hairpin/MI0006335	1,38	0,045
	BM	MIR193B	hsa-miR-193B	https://mirbase.org/hairpin/MI0003137	1,37	0,044
	BM	MIR4423	hsa-miR-4423	https://mirbase.org/hairpin/MI0016760	1,33	0,033
	BM	MIR3140	hsa-miR-3140	https://mirbase.org/hairpin/MI0014163	1,30	0,005
	BM	MIR320C2	hsa-miR-320c-2	https://mirbase.org/hairpin/MI0008191	1,29	0,038
	BM	MIR181A1	hsa-miR-181a-1	https://mirbase.org/hairpin/MI0000289	1,25	0,044
	BM	MIR618	hsa-miR-618	https://mirbase.org/hairpin/MI0003632	1,24	0,043
Down	BM	MIR124-2	hsa-miR-124-2	https://mirbase.org/hairpin/MI0000444	-1,28	0,022
	BM	MIR562	hsa-miR-562	https://mirbase.org/hairpin/MI0003568	-1,29	0,025
	BM	MIR548AM	hsa-miR-548am	https://mirbase.org/hairpin/MI0016904	-1,29	0,036
	BM	MIR23C	hsa-miR-23c	https://mirbase.org/hairpin/MI0016010	-1,29	0,022
	BM	MIR200C	hsa-miR-200c	https://mirbase.org/hairpin/MI0000650	-1,31	0,028
	BM	MIR4521	hsa-miR-4521	https://mirbase.org/hairpin/MI0016887	-1,34	0,042
	BM	MIR6735	hsa-miR-6735	https://mirbase.org/hairpin/MI0022580	-1,37	0,016
	BM	MIR1827	hsa-miR-1827	https://mirbase.org/hairpin/MI0008195	-1,38	0,030
	BM	MIR2117	hsa-miR-2117	https://mirbase.org/hairpin/MI0010636	-1,42	0,033
	BM	MIR4295	hsa-miR-4295	https://mirbase.org/hairpin/MI0015822	-1,43	0,039
	BM	MIR4518	hsa-miR-4518	https://mirbase.org/hairpin/MI0016884	-1,43	0,011
	BM	MIR134	hsa-miR-134	https://mirbase.org/hairpin/MI0000474	-1,45	0,037
	BM	MIR548AD	hsa-miR-548ad	https://mirbase.org/hairpin/MI0016770	-1,46	0,037
	BM	MIR3677	hsa-miR-3677	https://mirbase.org/hairpin/MI0016078	-1,47	0,027

	BM	MIR4296	hsa-miR-4296	https://mirbase.org/hairpin/MI0015823	-1,51	0,008
	BM	MIR3667	hsa-miR-3667	https://mirbase.org/hairpin/MI0016068	-1,52	0,015
	BM	MIR519A1	hsa-miR-519a-1	https://mirbase.org/hairpin/MI0003178	-1,52	0,006
	BM	MIR4739	hsa-miR-4739	https://mirbase.org/hairpin/MI0017377	-1,57	0,036
	BM	MIR130A	hsa-miR-130a	https://mirbase.org/hairpin/MI0000448	-1,57	0,042
	BM	MIR4710	hsa-miR-4710	https://mirbase.org/hairpin/MI0017344	-1,58	0,028
	BM	MIR520A	hsa-miR-520a	https://mirbase.org/hairpin/MI0003149	-1,68	0,002
	BM	MIR1913	hsa-miR-1913	https://mirbase.org/hairpin/MI0008334	-1,75	0,006
	BM	MIR544A	hsa-miR-544a	https://mirbase.org/hairpin/MI0003515	-1,78	0,017
	BM	MIR328	hsa-miR-328	https://mirbase.org/hairpin/MI0000804	-1,92	0,022
	BM	MIR3922	hsa-miR-3922	https://mirbase.org/hairpin/MI0016429	-1,92	0,036
	BM	MIR1587	hsa-miR-1587	https://mirbase.org/hairpin/MI0016905	-2,00	0,020
	BM	MIR191	hsa-miR-191	https://mirbase.org/hairpin/MI0000465	-2,09	0,027
	BM	MIR4740	hsa-miR-4740	https://mirbase.org/hairpin/MI0017378	-2,13	0,047
	BM	MIR4526	hsa-miR-4526	https://mirbase.org/hairpin/MI0016893	-2,36	0,028

Supplementary Table S1: miRNA genes up- and downregulated by heparin (≥ 1.2 or ≤ -1.2 fold) for each stromal cell tissue source ranked from highest to lowest.
