# Supplementary material for: Heparin Differentially Regulates the Expression of Specific miRNAs in Mesenchymal Stromal Cells
Source: Int J Mol Sci. 2024 Nov 23;25(23):12589. doi: 10.3390/ijms252312589 (PMC11641817; doi:10.3390/ijms252312589)
Supplement: Supplementary file 1 [file ijms-25-12589-s001.zip › Supplementary_TableS2.pdf]

**miRNA name**	**Specific forward primer sequence (5‘-3‘)**	**Unique reverse primer sequence (5‘-3‘)**
SNORD44	AACAAGCCTGGATGATGATAAGCAA	CAGTGCAGGGTCCGAGGT
SNORD48	AACAAGAGTGATGATGACCCCAG	CAGTGCAGGGTCCGAGGT
hsa-miR-3671	ATGCGCGCATCAAATAAGGACTA	CAGTGCAGGGTCCGAGGT
hsa-miR-4451	AAGAGCGTTGGTAGAGCTGAGG	CAGTGCAGGGTCCGAGGT
hsa-miR-1260B	AACAAGATCCCACCACTGCCA	CAGTGCAGGGTCCGAGGT
hsa-miR-191-3p	AACAAGGCTGCGCTTGGATTT	CAGTGCAGGGTCCGAGGT
hsa-miR-1827	AACACGTGTGAGGCAGTAGATTG	CAGTGCAGGGTCCGAGGT
hsa-miR-1587	AACAAGTTGGGCTGGGCTG	CAGTGCAGGGTCCGAGGT
hsa-miR-32-5p	ACGCCGTATTGCACATTACTAAG	CAGTGCAGGGTCCGAGGT
hsa-miR-326	AACAATCCTCTGGGCCCTTCC	CAGTGCAGGGTCCGAGGT
hsa-miR-455-3p	AACAGAGCAGTCCATGGGCATAT	CAGTGCAGGGTCCGAGGT
hsa-miR-31-5p	AACACGCTGCTATGCCAACA	CAGTGCAGGGTCCGAGGT
hsa-miR-199A-5p	AACCATGCCCAGTGTTCAGACTA	CAGTGCAGGGTCCGAGGT
hsa-miR-539-5p	AGGCGTGCGGAGAAATTATCC	CAGTGCAGGGTCCGAGGT
hsa-miR-504-3p	AAGAAGAGGGAGTGCAGGGC	CAGTGCAGGGTCCGAGGT
hsa-miR-1226-3p	AACAAGTCACCAGCCCTGTGT	CAGTGCAGGGTCCGAGGT
hsa-miR-4316	AACAAGGGTGAGGCTAGCTGG	CAGTGCAGGGTCCGAGGT
hsa-miR-3689	AAGGTTGTCTGGGAGGTGTGATA	CAGTGCAGGGTCCGAGGT
hsa-miR-3619	AACAAGTCAGCAGGCAGGC	CAGTGCAGGGTCCGAGGT
hsa-miR-218-5p	AACCGGTTGTGCTTGATCTAACC	CAGTGCAGGGTCCGAGGT
Poly(T)Adapter-Primer for cDNA synthesis (5‘-3‘)	CAGTGCAGGGTCCGAGGTCAGAGCCACCTGGGCAATTTTTTTTTTTVN<br>V = A, G or C, but not T, N = A, G, C or T	

Supplementary Table S2: miRNA primer sequences used for qRT-PCR.
